# Supplementary material for: Plasmid diversity among genetically related Klebsiella pneumoniae blaKPC-2 and blaKPC-3 isolates collected in the Dutch national surveillance
Source: Sci Rep. 2020 Oct 8;10:16778. doi: 10.1038/s41598-020-73440-2 (PMC7546619; doi:10.1038/s41598-020-73440-2)
Supplement: Supplementary file 5 — Supplementary Information. [file 41598_2020_73440_MOESM5_ESM.docx]

**Supplemental Figure 1. Resistome of *K. pneumoniae* *bla*_KPC-2_ and *bla*_KPC-3_ non-cluster isolates.** *K. pneumoniae* *bla*_KPC-2_ isolates were marked blue, and *K. pneumoniae* *bla*_KPC-3_ were marked magenta. *K. pneumoniae* *bla*_KPC-2_ and *bla*_KPC-3_ cluster isolates were indicated on the y-axis and AMR genes on the x-axis. The UPGMA clustering was based on the presence (black squares) and absence of AMR genes. All isolates analysed contained the *fosA, oqxA* and *oqxB* AMR genes and were not included in this figure. Antibiotic classes are indicated above the AMR genes with different colors. A dotted line marks the 85% cut off.

**Supplemental Figure 2. *K. pneumoniae* plasmid gene content (continued).** An UPGMA clustering was performed based on the plasmid DNA sequence for the determination of the genetic relation among the 22 plasmids. Similarity is indicated on the y-axis using a scale from 0 (not similar) to 100% (identical). A similarity of ≥85 to 100% is regarded as the same plasmid. The plasmids are indicated on the x-axis. The presence (black squares) and absence is indicated of annotated genes among the 22 plasmids of six TGS sequenced isolates. If a gene was present twice, blue squares were used and more than two, red squared were used. Colors indicated different clusters of genes with a specific function. The light grey area indicates gene specific content found in only one plasmid. In the UPGMA tree, large plasmids are indicated in red, medium plasmids in black and small plasmids in green color.

**Supplemental table 1. Predicted prophage sequences among *K. pneumoniae* chromosomes.**

**Supplemental table 2. Antimicrobial resistance data, sequence type data and accession numbers of raw reads deposited in the Sequence Read Archive of the isolates analyzed in this study.**
